# Loss of p53 Provokes NF-κB-Dependent Disruption of Nucleolar Cap and Nucleoplasmic Redistribution of Fibrillarin During Nucleolar Stress

**DOI:** 10.3390/biom16020296

**Published:** 2026-02-13

**Authors:** Takeru Torii, Mako Sumida, Atsushi Kobayashi, Toshiyuki Goto, Ryosuke Suzuki, Shin Kuwamoto, Wataru Nakajima, Wataru Sugimoto, Kohei Takeuchi, Yuma Tanaya, Masayuki Tera, Nobuyuki Tanaka, Hiroaki Hirata, Hisae Tateishi-Karimata, Takahito Nishikata, Miwako Kato Homma, Daisuke Miyoshi, Keiko Kawauchi

**Affiliations:** 1 Faculty of Frontiers of Innovative Research in Science and Technology (FIRST), Konan University, Kobe 650-0047, Hyōgo, Japan; grignard.tor2@gmail.com (T.T.); nicomako0531@gmail.com (M.S.); cobashi.2929@gmail.com (A.K.); toshiyuki.goto.2805@outlook.jp (T.G.); kuwa.jhy@gmail.com (S.K.); los.wrrrwve.56s2@gmail.com (W.S.); takeuchikohei0816@gmail.com (K.T.); tateishi@konan-u.ac.jp (H.T.-K.); nisikata@konan-u.ac.jp (T.N.); 2 Graduate School of Science, Technology and Innovation, Kobe University, Kobe 650-0047, Hyōgo, Japan; 3 Department of Molecular Oncology, Institute for Advanced Medical Sciences, Nippon Medical School, 1-1-5 Sendagi, Bunkyo-ku 113-8602, Tokyo, Japan; nakaji@nms.ac.jp (W.N.); nobuta@nms.ac.jp (N.T.); 4 Department of Biotechnology and Life Science, Tokyo University of Agriculture and Technology, 2-24-16 Naka-cho, Koganei 184-8588, Tokyo, Japan; s247867w@st.go.tuat.ac.jp (Y.T.); tera@go.tuat.ac.jp (M.T.); 5 Department of Life Science and Biotechnology, Kanazawa Institute of Technology, Hakusan 924-0838, Ishikawa, Japan; hirata@neptune.kanazawa-it.ac.jp; 6 Department of Biomolecular Sciences, Fukushima Medical University School of Medicine, Hikarigaoka, Fukushima 960-1295, Fukushima, Japan; mkhomma@fmu.ac.jp; 7 Southern TOHOKU Research Institute for Neuroscience, 7-115 Yatsuyamada, Koriyama 963-8563, Fukushima, Japan

**Keywords:** p53, NF-κB, nucleolar stress, fibrillarin, G-quadruplex, gene expression

## Abstract

Chemotherapeutic agents targeting ribosome biogenesis induce profound reorganization of nucleolar architecture, yet how the tumor suppressor p53 governs these structural responses remains unclear. Here, we show that loss of p53 leads to NF-κB-dependent disappearance of nucleolar caps induced by doxorubicin (DOXO). Under these conditions, fibrillarin (FBL), which is normally confined to the nucleolus, relocates to the nucleoplasm and forms foci that partially associate with G-quadruplex (G4) structures, non-canonical nucleic acid secondary structures enriched at transcriptionally active genomic regions. To examine whether this redistribution is linked to transcriptional changes, we integrated publicly available transcriptomic datasets and identified genes that were upregulated in p53-deficient cells under DOXO treatment and downregulated upon FBL depletion. Given that casein kinase 2 alpha (CK2α) is a nuclear binding partner of FBL, we further analyzed CK2α-dependent gene programs. This analysis revealed that a fraction of FBL-responsive genes overlapped with CK2α-dependent signatures and were enriched for promoter-proximal G4 structures. Among candidate regulators, the G4-binding transcription factor MAZ emerged as a potential mediator linking nucleoplasmic FBL and CK2α to G4-associated transcriptional regulation. Together, our findings identify a mechanism linking loss of p53 to G4-associated transcriptional reprogramming through nucleolar architectural disruption mediated by an FBL–CK2α–MAZ axis during DOXO treatment.

## 1. Introduction

The nucleolus is the principal site of ribosome biogenesis, coordinating ribosomal RNA (rRNA) transcription, the processing and modification of precursor rRNA, and the early steps of ribosomal subunit assembly [1,2,3]. The nucleolus forms through liquid–liquid phase separation (LLPS) and exhibits three-layered organization composed of the fibrillar center (FC), dense fibrillar component (DFC), and granular component (GC) [3,4]. rRNA transcription is mediated by RNA polymerase I and takes place at the interface between FC and DFC, whereas processing of precursor rRNA occurs within the DFC and the assembly of preribosomal particles proceeds in GC. Mammalian cells typically harbor one to several nucleoli, and their number and structural features fluctuate with proliferative and metabolic demands [3].

Disruption of ribosome biogenesis triggers rapid reorganization of nucleolar components, including segregation of FC and DFC to the periphery and the formation of nucleolar caps [5,6,7]. Concurrently, NPM1 (also known as nucleophosmin, NO38, numatrin or B23) and ribosomal proteins such as RPL5 and RPL11 relocate to the nucleoplasm, where they bind to the E3 ubiquitin ligase MDM2 its binding to p53, thereby, stabilizing and activating p53 [8,9,10,11]. Engagement of this pathway results in cell-cycle arrest, senescence, or apoptosis called the nucleolar surveillance pathway [12,13].

Cancer cells maintain ribosome biogenesis at persistently high levels to support their rapid proliferation, making rRNA synthesis highly sensitive to therapeutic inhibition [14]. Accordingly, a broad range of chemotherapeutic agents exert their cytotoxic effects by disturbing nucleolar function [12,15]. Among them, DNA-damaging agents such as doxorubicin (DOXO), cisplatin, and mitomycin C suppress rRNA transcription indirectly through activation of DNA damage-responsive signaling pathways, whereas RNA polymerase I inhibitors such as actinomycin D, bind directly to GC-rich regions of rDNA and rapidly halt transcription of rRNA [10]. Despite their distinct mechanisms, both classes of agents disrupt ribosome biogenesis, induce nucleolar reorganization, and robustly activate the nucleolar surveillance pathway [12,13]. Because the nucleolar surveillance pathway operates in a strongly p53-dependent manner [13], cancers harboring p53 mutations—approximately half of all human malignancies—often fail to mount an adequate response to nucleolar stress [16,17]. This impaired response is thought to contribute to their reduced sensitivity to chemotherapy.

Recent studies have demonstrated that nucleolar stress induces extensive structural reorganization within the nucleolus, accompanied by the relocalization of major nucleolar proteins such as NPM1, nucleolin (NCL), and fibrillarin (FBL) from the nucleolus to the nucleoplasm and, in some cases, to the cytoplasm [9,18,19,20,21,22]. After relocalization, these proteins participate in diverse stress-responsive pathways, including apoptosis [8,12]. Increasing evidence indicates that nuclear structural elements actively contribute to nucleolar cap organization. Nuclear actin filaments facilitate cap formation, whereas depletion of the nuclear lamina component lamin B2 results in cap disruption [21,23]. These observations underscore the tight coordination between nucleolar architecture and transcriptional and chromatin states. Although p53 has been implicated in promoting nuclear actin filament assembly [24] while repressing lamin B2 expression [25], the mechanisms by which p53 regulates these architectural responses of the nucleolus during cellular stress remain unclear.

FBL predominantly localizes to the DFC of the nucleolus and plays essential roles in ribosome biogenesis. It mediates 2′-O-methylation and processing of precursor rRNA as a core component of box C/D small nucleolar ribonucleoproteins, and also contributes to rRNA transcription through histone H2A Q104 methylation-dependent regulation [26,27]. Structurally, FBL consists of an N-terminal glycine–arginine-rich (GAR) domain and a C-terminal methyltransferase (MTase) domain [28,29]. The GAR domain is required for nucleolar localization and exhibits self-association properties that support LLPS underlying DFC organization [30]. In addition to these nucleolar functions, recent studies have demonstrated that FBL also participates in the maturation of splicing-related RNAs and the transcriptional regulation of specific genes within the nucleoplasm [31], and that its dysregulation contributes to enhanced proliferation and malignant phenotypes in cancer cells [32].

In this study, we show that loss of p53 function is associated with alterations in DOXO-induced nucleolar cap organization in an NF-κB–dependent manner and relocalization of FBL to the nucleoplasm. Nucleoplasmic FBL shows partial association with G-quadruplexes (G4s), which are noncanonical nucleic acid conformations that are known to function as transcriptional hubs [33]. Integrative analyses further show that relocalization of FBL toward G4-rich loci is associated with transcriptional alterations characteristic of cells with loss of p53 function, including upregulation of cell cycle-related genes such as *PRC1*, thereby linking changes in nucleolar architecture to G4-associated transcriptional regulation.

## 2. Materials and Methods

### 2.1. Cell Culture and Materials

Human colon cancer p53^+/+^ (WT) and p53^−/−^ (KO) HCT116 cells were kindly provided by Dr. Bert Vogelstein (Johns Hopkins University, Baltimore, MD, USA). Human breast cancer MCF-7 cells and human embryonic kidney HEK293T cells are obtained from the American Cells Type Culture Collection (Manassas, VA, USA). Cells were cultured in Dulbecco’s modified Eagle’s medium (Nissui Pharmaceutical Co., Ltd., Tokyo, Japan) supplemented with 10% fetal bovine serum and 1% penicillin/streptomycin. Doxorubicin (DOXO) and Actinomycin D (ActD) were purchased from Calbiochem (La Jolla, CA, USA) and Fujifilm (Tokyo, Japan), respectively.

### 2.2. Retrovirus Infection

The retrovirus infections were performed as described previously [34,35]. Briefly, retroviruses encoding short hairpin RNAs (shRNAs) targeting human *TP53* or *RELA* were generated by cloning the following target sequences into the pSuper retro puro vector (Oligoengine, Seattle, WA, USA): human *TP53*, 5′-GACTCCAGTGGTAATCTAC-3′; human *RELA*, 5′-GGGATGAGATCTTCCTACTGT-3′. Infected cells were selected using 1.5 µg/mL puromycin for 2–3 days.

### 2.3. Immuno-Fluorescence

The cells were fixed with 4% paraformaldehyde (PFA) in phosphate-buffered saline (PBS) for 30 min at room temperature (RT) and permeabilized with 0.1% Triton X-100 for 15 min at RT for immunofluorescence. After blocking with 2% BAS in PBS, the cells were incubated with His-tagged Fab fragment BG4 antibody against the G-quadruplex overnight at 4 °C and washed three times with ice-cold PBS. The cells were then incubated overnight at 4 °C with anti-His (3D5; Absolute Antibody, Redcar, UK, ab206498, Cambridge, MA, USA) bound to BG4, anti-fibrillarin (C13C3: #2639, Cell Signaling Technology, Danvers, MA, USA), anti-B23 (0412: sc-47725, Santa Cruz Biotechnology, Dallas, TX, USA), SC-35 (ab11826; Abcam, Cambridge, UK) antibodies, or CK2α monoclonal antibody (clone 10B2) followed by washing with ice-cold PBS. Purification and validation of the BG4 antibody were described previously [36]. Purification and validation of the CK2α monoclonal antibody (clone 10B2) have been reported previously [37,38] and in Patent US20240376227A1.

The cells were incubated with Alexa Fluor 488 or 647-conjugated goat anti-mouse IgG (Invitrogen, Waltham, MA, USA) and Alexa Fluor 488, 546, or 647-conjugated goat anti-rabbit IgG (Invitrogen, Waltham, MA, USA) for 1 h at RT or overnight at 4 °C. After washing with ice-cold PBS, the cells were fixed again with 4% PFA in PBS and subsequently stained with 4′,6-diamidino-2-phenylindole (DAPI; Vector Laboratories, Burlingame, CA, USA). Images were acquired using a confocal microscope (A1R HD25, Nikon, Tokyo, Japan, RRID: SCR_020317) and analyzed using the Fiji software version 1.53t (National Institutes of Health, Bethesda, MD, RRID: SCR_002285). The acquired images for FBL, SC35, and G4 were deconvoluted using the NIS Elements AR software, version 5.11.01.

### 2.4. Immunoblot Analysis

Immunoblot analysis was performed as described previously [1]. Briefly, cells were solubilized with lysis buffer (50 mM Tris pH 7.4, 150 mM NaCl, 1% Triton X-100, 1% SDS, 10 mM EDTA, 1 mM Na_3_VO_4_, 10 mM NaF, and protease inhibitor cocktail (Nacalai Tesque, Kyoto, Japan), sonicated and centrifuged at 20,000× *g* for 15 min. The supernatants were subjected to sodium dodecyl sulfate-polyacrylamide gel electrophoresis (SDS-PAGE). Anti-p53 mouse monoclonal (DO-1; Santa Cruz Biotechnology, Dallas, TX, USA), anti-RELA/p65 mouse monoclonal (F-6; Santa Cruz Biotechnology, Dallas, TX, USA), anti-fibrillarin (C13C3: #2639, Cell Signaling Technology, Danvers, MA, USA), and anti-α-tubulin mouse monoclonal (DM1A; Santa Cruz Biotechnology, Dallas, TX, USA) antibodies were used.

### 2.5. Quantitative Real-Time PCR

Total RNA was isolated and purified using an ISOSPIN Cell & Tissue RNA Kit (NIPPON GENE Co., Ltd., Tokyo, Japan). For quantitative real-time PCR (qRT-PCR), cDNA was synthesized using the PrimeScript RT Reagent Kit (Perfect Real Time) (Takara Bio Inc., Shiga, Japan). qRT-PCR was performed with THUNDERBIRD SYBR qPCR Mix (Toyobo, Osaka, Japan) on a CFX Duet Real-Time PCR system (Bio-Rad Laboratories, Inc., Hercules, CA, USA) under the following conditions: 95 °C for 1 min, followed by 40 cycles of 95 °C for 15 sec and 60 °C for 1 min. The following primers were used: human *PRC1*, forward 5′-ATAGCCAGGAGCAGAGACAAGC-3′ and reverse 5′-AACCGCACAATCTCAGCATCGTG-3′; human *FBXO5*, forward 5′-GATATTCTCAGCGAACTCTTTCGA-3′ and reverse 5′-GGATCTTCTTCCAAGTTGTGCTC-3′; human *CCNB1*, forward 5′-GACCTGTGTCAGGCTTTCTCTG-3′ and reverse 5′-GGTATTTTGGTCTGACTGCTTGC-3′; human *PPIA*, forward 5′-GGCAAATGCTGGACCCAACACA-3′ and reverse 5′-TGCTGGTCTTGCCATTCCTGGA-3′Relative mRNA expression levels were normalized to human *PPIA* and calculated relative to the control.

### 2.6. Statistical Analysis

Statistical comparisons were performed using Welch’s two-sided *t*-test. *, **, and *** indicate *p* < 0.05, *p* < 0.01, and *p* < 0.001, respectively.

## 3. Results

### 3.1. Loss of p53 Disrupts Nucleolar Cap Integrity Under Doxorubicin Treatment

Doxorubicin (DOXO), a DNA-damaging agent, is known to induce nucleolar cap formation [12,15]. Recent studies have shown that nuclear actin filaments contribute to this process through the LINC complex, and that nuclear envelope infoldings also participate in nucleolar reorganization associated with cap formation [23]. Based on our previous finding that loss of p53 promotes the formation of nuclear actin filaments upon DOXO treatment [24], we hypothesized that p53 might influence nucleolar cap formation. To test this possibility, we analyzed the DOXO-induced reorganization of nucleolar components by examining the localization of FBL and NPM1, which predominantly localize to the DFC and GC, respectively, in WT and p53 KO HCT116 cells.

**Figure 1:**
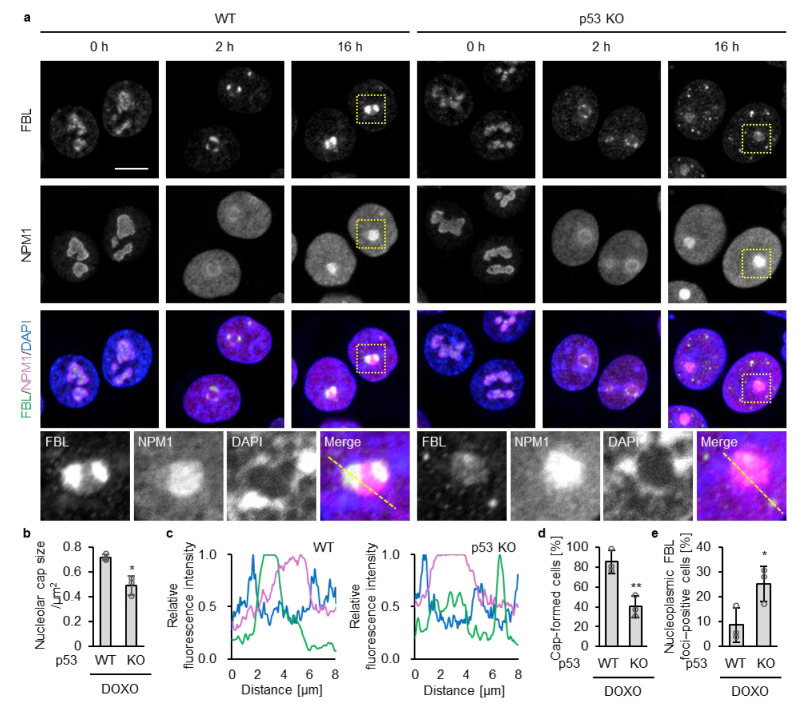
Nucleolar caps are fewer and nucleoplasmic FBL foci are more abundant in p53 KO cells than in WT cells upon the doxorubicin treatment. (**a**) WT and p53 KO HCT116 cells were treated with or without DOXO (1 μg/mL) for 2 or 16 h. Confocal images of FBL (green), NPM1 (magenta), and DAPI-stained DNA (blue). The scale bar is 10 µm. Yellow dotted boxes indicate the enlarged areas. (**b**) FBL enrichment in nucleolar caps was quantified in DOXO-treated cells (2 h). For each cell, the size of nucleolar caps indicated by FBL signals was measured (140–160 nucleolar caps per sample). (**c**) Line plots of FBL, NPM1, and DAPI fluorescence intensities along the yellow dotted lines in (**a**) from DOXO-treated cells (16 h). The intensity values were normalized to the maximum value of each fluorescence. (**d**) The proportion of nucleolar cap–positive cells was quantified from confocal images of DOXO-treated cells (16 h). Cap-positive cells were defined as cells showing peripheral accumulation of FBL at NPM1-positive nucleoli, whereas cells lacking such peripheral accumulation or showing higher nucleoplasmic than nucleolar FBL intensity were classified as cap-negative. (**e**) The proportion of nucleoplasmic FBL–positive cells was quantified from confocal images of DOXO-treated cells (16 h); cells containing five or more nucleoplasmic FBL foci (≥5 foci) were considered positive. (**d**,**e**) Quantification was performed in cells with NPM1-positive nucleoli (140–160 cells per sample). (**b**,**d**,**e**) Values are presented as mean ± SD from three independent experiments. Statistical significance was assessed using an unpaired two-sided *t*-test with Welch’s correction. * and ** indicate *p* < 0.05 and *p* < 0.01, respectively.

Under untreated conditions, WT and KO cells exhibited irregularly shaped nucleoli in which FBL and NPM1 localized, as expected, to the inner and outer regions of the nucleolus, respectively (Figure 1a). At 2 h treatment of DOXO, nucleoli in both WT and p53 KO HCT116 cells became rounded, accompanied by redistribution of FBL and NPM1 toward the nucleolar periphery. At this time point, NPM1 concurrently exhibited a diffuse distribution throughout the nucleoplasm, as previously reported [12]. Although FBL redistributed toward the nucleolar periphery in both genotypes, the intensity of peripheral FBL enrichment was substantially weaker in p53 KO cells than in WT cells (Figure 1b).

At 16 h of DOXO treatment, FBL remained at the nucleolar cap in WT cells (Figure 1a,c). In contrast, in p53 KO cells, nucleoplasmic FBL foci were more frequently observed, and FBL localization at the nucleolar cap was lower than that in WT cells (Figure 1a,c), despite comparable total FBL protein levels between WT and p53-deficient cells (Supplementary Figure S1). Quantitative analyses confirmed a significant increase in nucleoplasmic FBL foci in p53 KO cells compared with WT cells following DOXO treatment (Figure 1d,e). In MCF-7 cells bearing wild-type p53 treated with DOXO for 16 h, increased nucleoplasmic FBL foci and reduced nucleolar cap–associated FBL were observed only upon p53 depletion by shRNA, consistent with a p53 loss–dependent alteration in FBL dynamics (Supplementary Figure S2).

Further, to determine whether this effect is attributable to loss of p53 function, we examined the impact of a dominant-negative p53 mutant. A p53 mutant harboring the R175H mutation together with L22Q and W23S substitutions, which impair both DNA-binding–dependent and transcriptional activation functions of p53 [39,40], was ectopically expressed in WT HCT116 cells. An EGFP expression vector was co-transfected, and EGFP-positive cells were used to identify cells expressing the p53 mutant (Supplementary Figure S3a). Following 16 h of DOXO treatment, EGFP-positive cells expressing the p53 mutant exhibited significantly reduced nucleolar cap size compared with EGFP-positive control cells transfected with EGFP alone (Supplementary Figure S3b,c). Although nucleoplasmic FBL signals were occasionally observed in control cells, nucleoplasmic FBL signals were more likely to be prominent in cells expressing the p53 mutant. Taken together, these results suggest that loss of p53 function facilitates disruption of DOXO-induced nucleolar caps.

### 3.2. NF-κB/RelA Plays a Role in Nucleolar Cap Disruption in p53-Deficient Cells Under Doxorubicin Treatment

**Figure 2:**
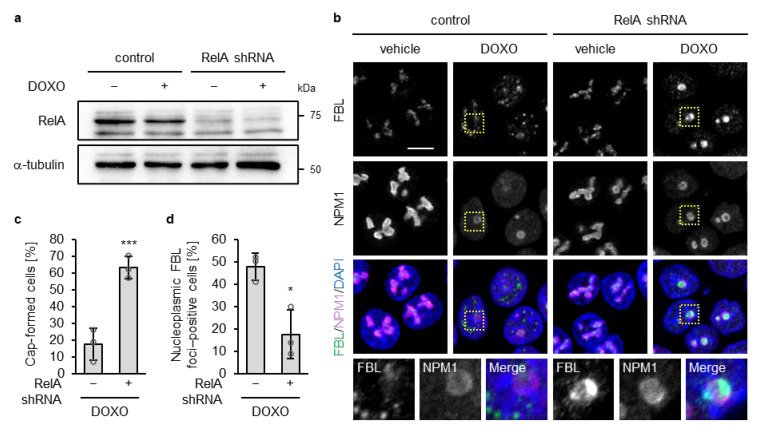
RelA knockdown increased nucleolar cap formation and decreased nucleoplasmic FBL foci in DOXO-treated p53 KO cells. (**a**,**b**) p53 KO HCT116 cells expressing control or RelA shRNA were treated with or without DOXO (1 µg/mL) for 16 h. (**a**) Cell lysates were subjected to immunoblot analysis with antibodies against RelA and α-tubulin as a loading control. (**b**) Confocal images of FBL (green), NPM1 (magenta), and DAPI-stained DNA (blue). The scale bar is 10 µm. Yellow dotted boxes indicate the expansion area. (**c**) The proportion of nucleolar cap–positive cells was quantified from confocal images of DOXO-treated cells. Cap-positive cells were defined as cells showing peripheral accumulation of FBL at NPM1-positive nucleoli, whereas cells lacking such peripheral accumulation or showing higher nucleoplasmic than nucleolar FBL intensity were classified as cap-negative. (**d**) The proportion of nucleoplasmic FBL–positive cells was quantified from confocal images of DOXO-treated cells. Cells containing five or more nucleoplasmic FBL foci (≥5 foci) were considered positive. (**c**,**d**) Quantification was performed in cells with NPM1-positive nucleoli (140–150 cells per sample). Values are presented as mean ± SD from three independent experiments. Statistical significance was assessed using an unpaired two-sided *t*-test with Welch’s correction. * and *** indicate *p* < 0.05 and *p* < 0.001, respectively.

Loss of p53 constitutively activates NF-κB signaling, and this activation is further enhanced by DOXO treatment [41]. Although NF-κB is well known as a transcription factor, nucleolar NF-κB—particularly RelA (also known as p65, a core component of the NF-κB complex)—has been reported to modulate nucleolar structure and promote its disassembly under specific stresses such as aspirin treatment and UV irradiation [42,43], we examined whether RelA contributes to disruption of the nucleolar caps in p53-deficient cells upon DOXO treatment. To test this, we performed shRNA-mediated knockdown of RelA in p53 KO HCT116 cells using a retroviral system (Figure 2a). Under the untreated condition, RelA depletion had little to no effect on the localization of FBL or NPM1, and nucleolar morphology remained indistinguishable from that in control shRNA–expressing p53 KO cells, indicating that RelA knockdown alone did not perturb nucleolar organization (Figure 2b). At 16 h of DOXO treatment, RelA depletion in p53 KO cells promoted nucleolar cap formation and suppressed the accumulation of FBL in the nucleoplasm (Figure 2b). Quantitative analyses confirmed a significant increase in the proportion of nucleolar cap–positive cells and a concomitant decrease in nucleoplasmic FBL–positive cells following RelA depletion under DOXO treatment (Figure 2c,d). These findings support a role for NF-κB/RelA in promoting nucleolar cap disruption in p53-deficient cells in response to DOXO treatment.

### 3.3. NF-κB/RelA Plays a Role in Nucleolar Cap Disruption in p53-Deficient Cells Under Actinomycin D Treatment

**Figure 3:**
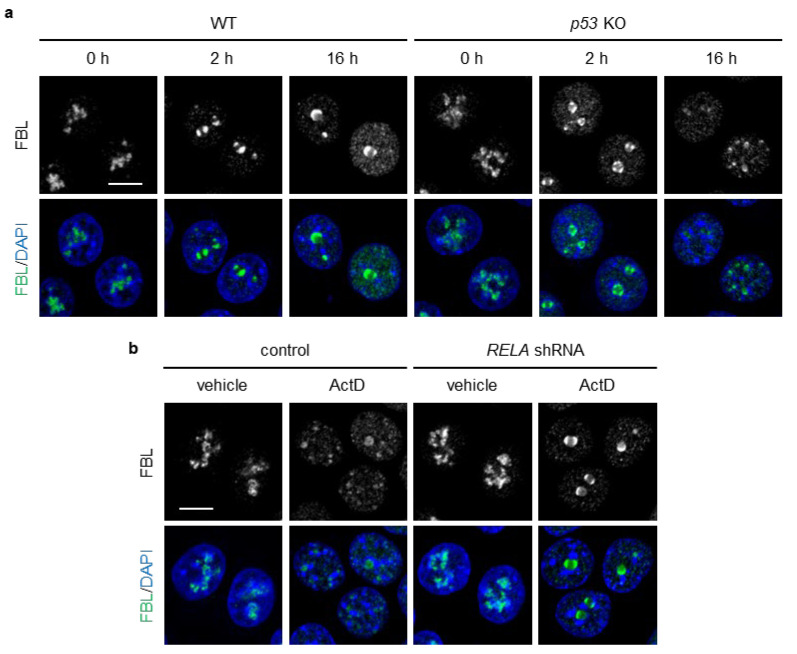
RelA knockdown suppressed ActD-induced nucleolar cap disruption and nucleoplasmic FBL localization in p53 KO cells. (**a**) WT and p53 KO HCT116 cells were treated with or without ActD (20 ng/mL) for 16 h. (**b**) p53 KO HCT116 cells expressing control or RelA shRNA were treated with or without ActD (20 ng/mL) for 16 h. (**a**,**b**) Confocal images of FBL (green) and DAPI-stained DNA (blue). The scale bar is 10 µm.

Actinomycin D (ActD) induces the formation of nucleolar caps by directly inhibiting rRNA transcription [12]. ActD has been reported not to cause nucleolar translocation of RelA [44], while it remains unclear whether DOXO triggers nucleolar translocation of RelA. We reasoned that ActD would enable us to evaluate whether loss of p53 leads to nucleolar cap disruption under conditions in which the influence of nucleolar NF-κB could be excluded. At 2 h of ActD treatment, both WT and p53 KO HCT116 cells formed nucleolar caps (Figure 3a). At 16 h of ActD treatment, WT cells maintained well-organized caps with FBL remaining at the nucleolar periphery, whereas p53 KO cells displayed dispersed nucleoplasmic FBL foci and a marked decrease in cap-positive cells (Figure 3a). NPM1 accumulated in the nucleoplasm to a similar extent in both WT and p53 KO cells. RelA knockdown increased the formation of nucleolar caps in p53 KO cell (Figure 3b). These results suggest that p53 plays an integral role in preserving nucleolar cap structures and retaining FBL within the caps in DOXO- or ActD- treated cells by suppressing NF-κB activation. Importantly, because ActD does not induce nucleolar translocation of RelA [44], nucleoplasmic NF-κB, rather than nucleolar NF-κB, may be involved in nucleolar cap disruption in p53 KO cells.

### 3.4. Identification of Genes Associated with Nucleoplasmic FBL in DOXO-Treated p53-Deficient Cells

**Figure 4:**
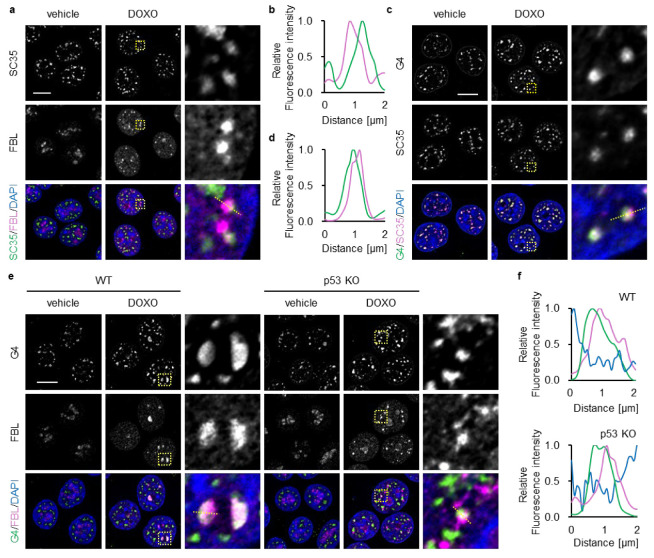
Spatial relationship of nucleoplasmic FBL foci with nuclear speckles and G4 foci. (**a**–**f**) WT (**e**,**f**) and p53 KO (**a**–**f**) HCT116 cells were treated with or without DOXO (1 μg/mL) for 16 h. (**a**) Confocal images of SC35 (green), FBL (magenta), and DAPI-stained DNA (blue). (**b**) Line plots of SC35 and FBL fluorescence intensity along the yellow dotted lines in (**a**). (**c**) Confocal images of G4 (green), SC35 (magenta), and DAPI-stained DNA (blue). (**d**) Line plots of G4 and SC35 fluorescence intensity along the yellow dotted lines in (**c**). (**e**) Confocal images of G4 (green), FBL (magenta), and DAPI-stained DNA (blue). (**f**) Line plots of G4 and FBL fluorescence intensity along the yellow dotted lines in (**e**). (**a**,**c**,**e**) The scale bar is 10 µm. Yellow dotted boxes indicate the expansion area. (**b**,**d**,**f**) The intensity values were normalized to the maximum value of each fluorescence.

FBL is implicated in the maturation of splicing-related RNAs and the transcriptional regulation of specific genes within the nucleoplasm [31,45]. Nuclear speckles, which function as hubs for RNA splicing and transcription, are formed through LLPS in close proximity to transcriptionally active chromatin [46]. Based on these observations, we hypothesized that the nucleoplasmic FBL observed in p53-deficient cells upon 16 h of DOXO treatment may localize to nuclear speckles. To evaluate this issue, we performed co-immunostaining for FBL and SC35, a nuclear-speckle marker [47]. Under untreated conditions, SC35 showed its characteristic punctate distribution throughout the nucleoplasm, and FBL remained confined to the nucleolus in p53 KO cells (Figure 4a). At 16 h of DOXO treatment, p53 KO cells displayed a complete collapse of nucleolar caps, which was accompanied by widespread nucleoplasmic redistribution of FBL. Notably, a subset of these nucleoplasmic FBL foci appeared closely apposed to SC35 foci, and line-intensity profiles indicated partial overlap between the two signals (Figure 4a,b), suggesting that ectopic FBL exhibits limited spatial association with speckle-linked regions in p53-deficient cells.

Nuclear speckles frequently reside near chromatin regions enriched in G4 [48,49]—noncanonical nucleic acid conformations that serve as regulatory hubs for transcription [33]. Therefore, we next examined the spatial relationship between FBL and G4. To visualize G4s, we used BG4, a widely used antibody that specifically recognizes folded G4 structures [50]. SC35 and G4 signals showed partial proximity even in both untreated and untreated p53 KO cells (Figure 4c,d), suggesting spatial coupling between speckles and G4-rich chromatin. At 16 h of DOXO treatment, G4 signals were concentrated at FBL-containing nucleolar caps in WT cells (Figure 4e,f). In contrast, p53 KO cells exhibited markedly diminished nucleolar G4 signals and numerous nucleoplasmic G4 foci. A subset of these G4 foci overlapped with, or were positioned adjacent to, dispersed nucleoplasmic FBL foci, indicating that DOXO induced a coordinated redistribution of both FBL and G4 toward shared nuclear domains under p53-deficient conditions. DOXO-treated p53-deficient cells show prominent redistribution of FBL from the nucleolus to the nucleoplasm, raising the possibility that nucleoplasmic FBL contributes to associated with DOXO-induced nucleolar stress.

**Figure 5:**
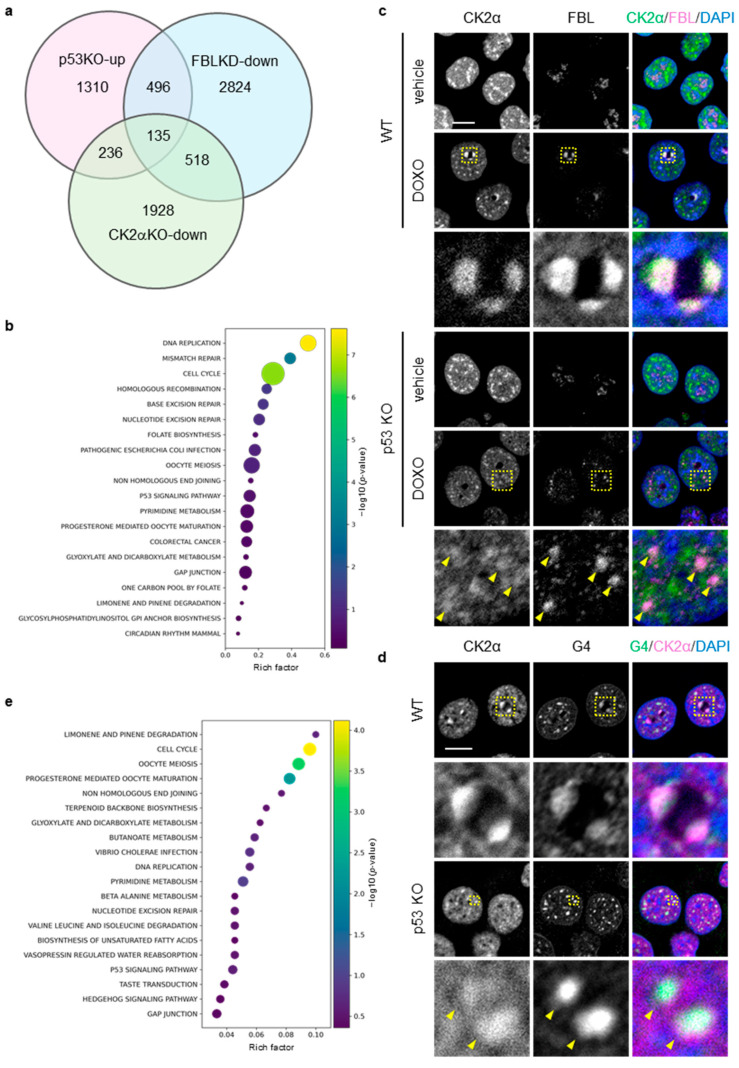
Identification of FBL-responsive gene sets and spatial association between FBL and nuclear CK2α under nucleolar stress in p53-deficient cells. (**a**) Venn diagram showing the overlap among genes upregulated in p53 KO HCT116 cells compared with WT cells under doxorubicin (DOXO) treatment, genes downregulated upon FBL knockdown in mitomycin C–treated HCT116 cells, and genes downregulated upon CK2α knockout in U937 cells (see Supplementary Material and Methods for details). (**b**) KEGG pathway enrichment analysis of the FBL-responsive gene set (630 genes), defined as genes upregulated in p53 KO HCT116 cells relative to WT cells under DOXO treatment and downregulated upon FBL knockdown. Bubble size indicates the number of genes associated with each pathway, and bubble color represents statistical significance expressed as −log10 (*p*-value). The *x*-axis shows the rich factor. (**c**,**d**) WT and p53 KO HCT116 cells were treated with or without DOXO (1 μg/mL) for 16 h. The scale bar is 10 µm. Yellow dotted boxes indicate the expansion area. (**c**) Confocal images of CK2 (green), FBL (magenta), and DAPI-stained DNA (blue). (**d**) Confocal images of G4 (green), CK2α (magenta), and DAPI-stained DNA (blue). (**e**) KEGG pathway enrichment analysis was performed for 135 genes downregulated upon CK2α knockout within the FBL-responsive gene set. Bubble size represents the number of overlapping genes, and bubble color indicates −log10 (*p*-value). The *x*-axis shows the rich factor. Yellow arrowheads indicate representative nucleoplasmic foci corresponding to redistributed FBL.

Because mitomycin C treatment induces nucleoplasmic relocalization of FBL in HCT116 cells [45], we focused on genes common to those downregulated by FBL knockdown under this condition (GSE205366) and those upregulated in p53 KO cells under DOXO treatment (GSE42368). This approach identified 630 shared genes, excluding FBL itself (Figure 5a; File S1). In this study, these genes were defined as FBL-responsive genes, representing a gene set that may be transcriptionally regulated by nucleoplasmic FBL in the context of p53 deficiency.

KEGG enrichment analysis revealed that FBL-responsive genes were enriched in pathways related to DNA replication, cell cycle, base excision repair (BER), mismatch repair (MMR), homologous recombination (HR), and nucleotide excision repair (NER) (Figure 5b).

Casein kinase 2 alpha (CK2α) is a kinase associated with anticancer drug responses and tumor progression in cancer cells [51,52]. Given that FBL interacts with nuclear CK2α and participates in transcriptional regulation [37], we next analyzed the spatial relationship among CK2α, FBL, and G4 structures under DOXO treatment by immunofluorescence. CK2α formed discrete foci in both the nucleoplasm and the nucleolus, and these foci partially colocalized with FBL within the nucleolus in both WT and p53 KO cells (Figure 5c). At 16 h of DOXO treatment, CK2α and FBL colocalized at nucleolar caps in WT cells, whereas in p53 KO cells their colocalization was predominantly observed in the nucleoplasm. Under the same conditions, CK2α and G4 foci colocalized at both nucleolar caps and in the nucleoplasm in WT cells, while in p53 KO cells their colocalization was restricted to the nucleoplasm (Figure 5d). Together, these observations indicate a close spatial relationship among CK2α, FBL, and G4 structures in the nucleoplasm under DOXO treatment.

Based on these findings, we next sought to identify CK2α-dependent genes within the 630 FBL-responsive genes, focusing on those potentially regulated by FBL in a G4-dependent manner. Using the data obtained from CK2α knockout (CK2α KO) U937 cells (GSE217776), we identified genes that were downregulated compared with WT cells and found that 135 genes overlapped with the 630 FBL-responsive genes (Figure 5a; File S2). KEGG enrichment analysis of these 135 genes showed that, in particular, within this CK2α-dependent refined gene set, cell cycle emerged as a prominent enriched pathway, together with DNA replication and DNA repair–related pathways including non-homologous end joining (NHEJ) (Figure 5e).

To examine whether these 135 CK2α-dependent FBL-responsive genes are potentially associated with G4-mediated transcriptional regulation, we analyzed endogenous G4 formation around their transcription start sites (TSS ± 1 kb). Endogenous G4 datasets for the HepG2 and K562 cell lines were obtained from the Gene Expression Omnibus (GSE145090; HepG2_async_rep1-3.mult.6of9.bed and K562_async_rep1-3.mult.5of8.bed). Genes in which G4 formation was detected in at least one of these cell lines were considered to harbor sequences with the potential to form G4 structures in vivo. Using this criterion, 92 out of the 135 genes (~68%) showed evidence of G4 formation in their promoter-proximal regions (File S3). Together, these results suggest that a substantial fraction of CK2α-dependent genes within the FBL-responsive gene set harbor promoter G4 structures and are preferentially enriched in G4-associated transcriptional programs related to cell cycle regulation and DNA replication/repair.

**Figure 6:**
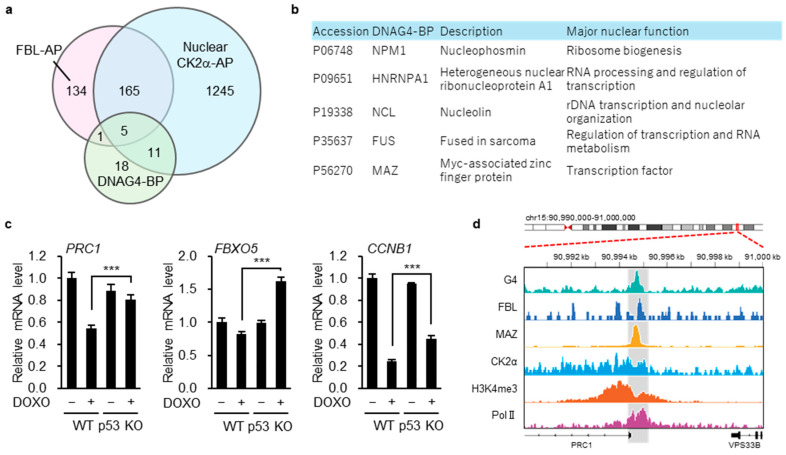
MAZ is a candidate transcription factor linking the CK2α–FBL axis to DNA G4-associated transcriptional regulation. (**a**) Venn diagram showing the overlap among FBL-associated proteins (FBL-AP), nuclear CK2α-associated proteins (CK2α-AP), and DNA G4-binding proteins (DNAG4-BP). (**b**) List of the five proteins commonly shared by FBL-AP, nuclear CK2α-AP, and DNAG4-BP. (**c**) p53 WT and p53 KO HCT116 cells were treated with or without DOXO (1 µg/mL) for 16 h. The expression of *PRC1*, *FBXO5*, or *CCNB1* was evaluated by quantitative real-time PCR. Each bar represents the mean ± S.D.; *n* = 4. Statistical significance was assessed using an unpaired two-sided *t*-test with Welch’s correction. *** indicate *p* < 0.001 (**d**) IGV tracks displaying G4 ChIP-seq (using the BG4 antibody; HepG2, GSM4474689), FBL ChIP-seq (MV-4-11, GSM8260719), MAZ ChIP-seq (HCT116, ENCFF622VZA), CK2α ChIP-seq (RPE, GSM7083688), H3K4me3 ChIP-seq (RPE, GSM7083690), and Pol II ChIP-seq (RPE, GSM7083692) across the *PRC1* locus. Data were obtained from ChIP-Atlas (https://chip-atlas.org/) and ENCODE (https://www.encodeproject.org/chip-seq/transcription_factor/) (accessed on 25 January 2026).

Based on the immunofluorescence results showing G4-associated localization (Figure 5d), we next sought to identify nuclear G4-binding proteins that interact with both FBL and CK2α. To identify such candidates, we first compiled a list of FBL-associated proteins from a publicly available mass spectrometry–based proteomics dataset (PXD034434) and nuclear CK2α-associated proteins from our own proteomics analysis (PXD040882). Comparison of these two protein sets revealed a substantial overlap (Figure 6a, File S4), with approximately 56% of the FBL-associated proteins also detected among nuclear CK2α-associated proteins, indicating a strong convergence between the two interactomes.

We then intersected this overlapping protein set with a curated list of established DNA G4-binding proteins compiled from multiple previous studies, as summarized in a recent review ([53]; File S5). This analysis identified five proteins—NPM1, hnRNP A1, NCL, FUS, and MAZ (Figure 6a,b).

Among these candidates, we focused on MAZ as a potential mediator linking the CK2α–FBL axis to G4-associated transcriptional regulation, as MAZ is a transcription factor known to associate with G4-forming promoter regions [54]. To this end, we examined whether MAZ target genes were enriched within the 135 CK2α-dependent genes identified from the FBL-responsive gene set. Using MAZ target gene annotations obtained from the ENCODE Transcription Factor Targets dataset via the Harmonizome database, we found that 131 of the 135 CK2α-dependent genes were annotated as MAZ targets in at least one publicly available dataset (File S6). From these genes, we selected *PRC1*, *FBXO5*, and *CCNB1*, as cell cycle-related genes that have been reported to be associated with poor prognosis across multiple cancer types [55,56,57,58]. qRT-PCR analysis confirmed that the expression levels of these genes were higher in p53 KO cells than in WT cells under DOXO treatment conditions (Figure 6c).

To assess the positional relationship between G4-forming sites and the CK2α–FBL–MAZ axis at the *PRC1* locus, we compared G4 ChIP-seq data obtained using the BG4 antibody from HepG2 cells (GSM4474689) with CK2α and FBL ChIP-seq data from RPE (GSM7083688) and MV-4-11 cells (GSM8260719), as well as MAZ ChIP-seq data from HCT116 cells (ENCFF622VZA), using IGV. As expected, a prominent G4 peak was detected at the *PRC1* promoter region, which was positionally coincident with peaks of CK2α and MAZ (Figure 5d). Although FBL ChIP-seq signals were broadly distributed across the genome, detectable local enrichment was observed in HCT116 cells at the same G4-positive promoter region. This region was further enriched for active chromatin features, including histone H3K4me3 and RNA polymerase II (Figure 6d).

**Figure 7:**
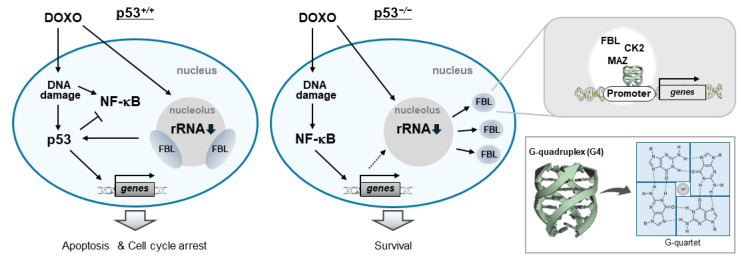
Proposed model for DOXO-induced nucleolar stress in which loss of p53 promotes NF-κB-dependent nucleolar cap disruption and FBL-mediated transcriptional remodelling. Solid arrows indicate activation or directional processes. T-shaped lines indicate inhibitory regulation. Dotted arrows represent proposed or indirect regulatory interactions. Downward arrows next to rRNA indicate reduced rRNA transcription under DOXO treatment.

Collectively, our results show that DOXO treatment in p53-deficient cells is associated with altered nucleolar organization and redistribution of FBL, accompanied by selective upregulation of genes involving *PRC1* gene. These genes share promoter-proximal G4 structures and exhibit positional coincidence with CK2α, MAZ, and FBL (Figure 7).

## 4. Discussion

In this study, we show that DOXO treatment induces relocalization of FBL from the nucleolus to the nucleoplasm, which is more pronounced in p53-deficient cells. Under these conditions, nucleoplasmic FBL is associated with transcriptionally active regions, G4-containing loci, and nuclear CK2α, extending the functional scope of FBL beyond its canonical nucleolar roles. Importantly, the reduced nucleolar cap formation observed in p53 KO cells is unlikely to simply reflect more advanced DOXO -induced cellular damage or accelerated cell death, as previous studies using HCT116 cells have shown that loss of p53 does not uniformly enhance DOXO -induced cytotoxic responses [59,60].

G4s are higher-order nucleic acid structures formed when four guanine bases assemble into a Hoogsteen hydrogen-bonded G-quartet and multiple quartets stack on top of each other [61,62]. Although the human genome contains an estimated seven hundred thousand potential G4-forming sequences [63], their formation in vivo is highly dynamic and depends on transcriptional and replicative states, chromatin context, and cellular stress [64,65,66]. Accumulating evidence indicates that G4 structures formed at promoter-proximal regions of active genes may serve as platforms that facilitate interactions with transcription-associated factors [64]. Recent studies have demonstrated that G4 structures promote the dynamic behavior of MAZ-containing nuclear condensates, thereby enhancing transcription of target genes such as *CCNB1* and contributing to oncogenic transcriptional programs [67].

We focused on CK2α, a nuclear binding partner of FBL, and identified MAZ as a transcription factor associating with both proteins, thereby linking nucleoplasmic FBL to G4-associated transcriptional regulation. CK2 is a ubiquitously expressed kinase composed of two catalytic α (or α′) subunits and two regulatory β subunits, forming a tetrameric holoenzyme, while the catalytic α subunits also retain kinase activity as monomers [68]. Notably, CK2 expression is frequently elevated in a wide range of cancers, including colorectal, breast, and lung cancers, and aberrant nuclear accumulation of CK2α has been associated with poor clinical outcomes [51,52]. Given that CK2 directly promotes MAZ activation [69], our findings suggest that interaction between CK2α and FBL under chemotherapeutic stress conditions, including DOXO treatment, may contribute to the formation of a transcriptionally relevant nucleoplasmic complex.

Recent studies have begun to elucidate molecular mechanisms by which FBL promotes transcription in a manner dependent on specific transcriptional regulators [31,45]. For example, FBL has been shown to enhance the DNA-binding activity of Y-box binding protein 1 (YBX1), thereby facilitating transcriptional activation of target genes [45]. In addition, FBL has been reported to cooperate with KH-type splicing regulatory protein (KHSRP) by binding to cis-regulatory elements, further supporting a role for nucleoplasmic FBL in transcriptional regulation through transcription factor-dependent mechanisms [31]. However, how FBL contributes to MAZ-dependent transcriptional activation remains unclear.

Sun et al. demonstrated that, in mitomycin C-treated HCT116 cells, FBL enhances *BRCA1* expression through YBX1 [45]. Notably, *BRCA1* expression is known to be suppressed by p53 [70]. Consistently with this notion, *BRCA1* was included in the set of 630 FBL-responsive genes identified in our analysis, although it was not retained within the more stringent subset of 135 CK2α-dependent FBL-responsive genes. Taken together, these observations indicate that FBL-mediated transcriptional regulation in cells with loss of p53 function is not governed by a single molecular pathway. Rather, nucleoplasmic FBL may regulate distinct subsets of genes through different transcriptional regulators, such as YBX1, KHSRP, and MAZ, depending on cellular context and stress conditions.

We found that alterations in nuclear architecture and transcription-associated processes observed in p53-deficient cells are closely linked to another major branch of the nucleolar stress response, the NF-κB signaling pathway (Figure 2 and Figure 3b). In this context, it has been reported that, in p53-deficient HCT116 cells treated with DOXO, suppression of NF-κB activity enhances DOXO-induced cytotoxicity [59]. NF-κB is a transcription factor activated by inflammatory cues such as TNFα as well as oncogenic signals including RAS, and is known to be constitutively activated in many cancers, where it contributes to tumor progression and chemoresistance [71,72,73]. Under these pathological contexts, p53 has been reported to suppress NF-κB signaling by inhibiting the activation of the IκB kinase (IKK) complex, thereby preventing phosphorylation and degradation of inhibitor of κB (IκB) and limiting the nuclear translocation and transcriptional activity of NF-κB [41]. Accordingly, loss of p53 has been associated with constitutive NF-κB activation. In colorectal cancer cells, increased expression of NF-κB target genes such as *TNFAIP3*, *PTGS2*, and *BCL2L1* has been reported [74,75,76,77]. Under DOXO treatment, the ATM–IKK cascade is activated, inducing nuclear translocation and transcriptional activation of NF-κB [78]. In contrast, atypical stimuli such as aspirin or UV-C trigger nucleolar translocation of NF-κB, resulting in suppression of its transcriptional activity and inducing nucleolar protein redistribution and nucleolar disassembly, including relocalization of NPM1 [43]. In this pathway, RelA enters the nucleus independently of IKK and subsequently undergoes modification by K63-linked ubiquitin chains. Unlike K48-linked chains that signal proteasomal degradation, K63-linked ubiquitin serves as a non-degradative signal that is recognized by the autophagy adaptor p62, which transports ubiquitinated RelA to the nucleolus [79,80]. Whether DOXO induces this p62-mediated nucleolar sequestration pathway remains unresolved. Importantly, NF-κB nucleolar translocation is not induced by TNFα or ActD treatment [44], suggesting that nucleolar targeting of NF-κB is highly stimulus-specific.

Taken together, the NF-κB-dependent nucleolar cap disruption and the mislocalization of FBL in p53-deficient cells under DOXO or ActD treatment are unlikely to be fully explained by p62-Ub-mediated nucleolar sequestration of NF-κB alone. Rather, our findings suggest that these phenotypes may be influenced, at least in part, by alterations in NF-κB-dependent transcriptional programs in the nucleoplasm. In this context, the loss of p53 may alleviate negative regulatory control over NF-κB, potentially altering the expression of NF-κB target genes in the nucleoplasm. Such alterations could, in turn, indirectly compromise nucleolar cap integrity and facilitate the aberrant redistribution of FBL. Although the present work does not fully resolve the mechanistic pathway, our data indicate that p53 loss and NF-κB-dependent transcriptional programs are involved in shaping nucleolar architecture during chemotherapeutic stress.

The mechanism underlying the formation of multiple nucleoplasmic FBL foci in p53-deficient cells remains unclear. DNA damage and nucleolar stress are known to induce reorganization of Cajal body (CB) components in a stress-dependent manner [81,82]. In particular, γ-irradiation shows p53-dependent differences in CB reorganization, whereas cisplatin induces a more general response with variable spatial patterns [82]. Given the association of FBL with CBs, such stress- and context-dependent CB reorganization may contribute to the redistribution of FBL under nucleolar stress.

## 5. Conclusions

We show that loss of p53 function is associated with altered organization of DOXO-induced nucleolar caps in an NF-κB/RelA-dependent context, accompanied by relocalization of FBL from the nucleolus to the nucleoplasm. In the nucleoplasm, FBL gains access to G4-enriched genomic regions, where its redistribution is associated with transcriptional responses characteristic of cells with loss of p53 function, including activation of cell cycle-related genes. These findings support a model in which loss of p53 function, NF-κB/RelA activity, and nucleoplasmic FBL cooperate to reshape G4-associated transcriptional programs, thereby creating a nuclear environment that favors stress adaptation and therapeutic resistance.

## Data Availability

Publicly available datasets analyzed in this study include microarray data (GSE42368), RNA-seq data for FBL knockdown (GSE205366), RNA-seq data for CK2α knockout (GSE217776), and BG4 ChIP-seq data (GSE145090), all obtained from the Gene Expression Omnibus (GEO). Proteomics datasets include PXD034434 (FBL-associated proteins) and PXD040882 (CK2α-associated proteins), available via ProteomeXchange and the jPOST repository. No new high-throughput sequencing or proteomics datasets were generated in this study. All supporting data are included in the article and its Supplementary Information.

## Supplementary Materials

The following supporting information can be downloaded at: https://www.mdpi.com/article/10.3390/biom16020296/s1, Supplementary Material and Method and Supplementary Figure S1: FBL protein levels in WT and *p53* KO HCT116 cells with or without DOXO treatment; Figure S2: The accumulation of nucleoplasmic FBL was induced by DOXO treatment in p53 knockdown MCF-7 cells; Figure S3: Dominant-negative p53 reduces nucleolar cap formation and increases nucleoplasmic FBL localization in DOXO-treated WT HCT116 cells; Figure S4: Original confocal images corresponding to Figure 4; Figure S5: Original uncropped images used for Western blot analysis: Torii_et_al_Supplementary_Information_Biomolecules; File S1 lists FBL-responsive genes identified in HCT116 cells under nucleolar stress (631 genes, including FBL); File S2 shows CK2α-dependent genes within the FBL-responsive gene set (135 genes); File S3 provides BG4 ChIP-seq enrichment data for these 135 CK2α-dependent FBL-responsive genes; File S4 summarizes proteins commonly identified in FBL- and nuclear CK2α-associated proteomes; File S5 contains a curated list of established DNA G-quadruplex–binding proteins compiled from multiple previous studies; File S6 lists MAZ target genes overlapping with the 135 CK2α-dependent FBL-responsive genes.

## Abbreviations

The following abbreviations are used in this manuscript:

FBL	Fibrillarin
G4	G-quadruplex
CK2α	Casein Kinase 2 alpha
MAZ	Myc-associated zinc finger protein
YBX1	Y-Box Binding Protein 1
rRNA	ribosomal RNA
LLPS	liquid–liquid phase separation
FC	fibrillar center
DFC	dense fibrillar component
GC	granular component
NPM1	nucleophosmin
NCL	nucleolin
DOXO	Doxorubicin
ActD	Actinomycin D
shRNA	short hairpin RNA
PFA	paraformaldehyde
PBS	phosphate-buffered saline
IDR	intrinsically disordered regions
RGG/RG	arginine–glycine–glycine/arginine–glycine
GAR	glycine–arginine–rich
ChIP	Chromatin immunoprecipitation
IκB	inhibitor of κB
