# Supplementary material for: Loss of p53 Provokes NF-κB-Dependent Disruption of Nucleolar Cap and Nucleoplasmic Redistribution of Fibrillarin During Nucleolar Stress
Source: Biomolecules. 2026 Feb 13;16(2):296. doi: 10.3390/biom16020296 (PMC12937983; doi:10.3390/biom16020296)
Supplement: Supplementary file 1 [file biomolecules-16-00296-s001.zip › Proof Sup Torii_et_al_Supplementary_Information_Biomolecules.pdf]

Supplementary Information

Loss of p53 Provokes NF-κB–Dependent Disruption of Nucleolar Cap and Nucleoplasmic Redistribution of FBL during Nucleolar Stress

Takeru Torii, Mako Sumida, Atsushi Kobayashi, Toshiyuki Goto, Ryosuke Suzuki, Shin Kuwamoto, Wataru Nakajima, Wataru Sugimoto, Kohei Takeuchi, Yuma Tanaya, Masayuki Tera, Nobuyuki Tanaka, Hiroaki Hirata, Hisae Tateishi-Karimata, Takahito Nishikata, Miwako Kato Homma, Daisuke Miyoshi, Keiko Kawauchi

Supplementary Information contains:

Supplementary Materials and Methods

Supplementary Figures S1–S5

Supplementary Reference

Supplementary Materials and Methods

Plasmid and transfection

The EGFP expression vector pEGFP-C1 (Clontech Laboratories, Mountain View, CA, USA) was used. The R175H mutation was introduced into the pEF-p53 L22Q/W23S construct described previously [1] by site-directed mutagenesis. The transfection reagent Lipofectamine 2000 (Thermo Fisher Scientific, Waltham, MA, USA) was used.

Integrated transcriptome analyses for defining the CK2α-dependent FBL-responsive gene set

Transcriptome data processing and gene set definition were performed as described below.

(1) Microarray processing

Non-normalized probe intensities from the HCT116 microarray dataset (GSE42368) were imported for eight arrays (WT control, WT doxorubicin 26 h, p53-KO control, and p53-KO doxorubicin 26 h; two biological replicates each). Probe intensities were log_2-transformed without an offset and quantile-normalized across all arrays using normalizeBetweenArrays in the limma package. A linear model was fitted using limma with separate coefficients for each experimental group, and contrasts were defined to compare KO_DOXO and WT_DOXO. Empirical Bayes moderation was applied using the eBayes function in limma. *P*-values were adjusted for multiple testing using the Benjamini–Hochberg method to control the false discovery rate (FDR), and genes with FDR < 0.05 were considered statistically significant. Normalized log_2 expression values were averaged within each condition (WT_CTRL, WT_DOXO, KO_CTRL, KO_DOXO), and KO–WT log_2 differences ("Δlog_2") were computed for untreated and DOXO-treated cells (Δlog_2_CTRL = KO_CTRL − WT_CTRL; Δlog_2_DOXO = KO_DOXO − WT_DOXO). When multiple probes mapped to the same gene symbol, the probe with the smallest adjusted *P*-value was retained as the representative at the gene level.

(2) RNA-seq processing for knockdown of FBL

RNA-seq data for FBL knockdown in mitomycin C-treated HCT116 cells (GSE205366) were processed using the limma–voom pipeline. Raw count data were normalized using the trimmed mean of M-values (TMM) method implemented in the edgeR package. Reads were aligned to the GRCh38 reference genome, gene-level counts were summarized, and low-abundance genes were filtered using filterByExpr in the edgeR package. Mean–variance trends were modeled using voom in the limma package, and differential expression between siControl and siFBL samples was assessed with limma. Linear modeling was performed within the limma framework, followed by empirical Bayes moderation. *P*-values were adjusted using the Benjamini–Hochberg method to control the FDR, and genes with FDR < 0.05 were considered significantly differentially expressed. The resulting moderated statistics provided the $\log_2$ fold change for *FBL* knockdown (logFC_siFBL).

(3) RNA-seq processing for knockout of *CSNK2A1* encoding CK2α

RNA-seq data from U937 cells with CK2α knockout (GSE217776; labeled as "CNK2A1 knockout" in the dataset, corresponding to *CSNK2A1* encoding CK2α) were analyzed using the limma–voom pipeline. The analysis included three biological replicates for CK2α knockout cells and four biological replicates for non-targeting control cells. Raw count data were TMM-normalized using the edgeR package, and low-abundance genes were filtered prior to downstream analysis. Mean–variance trends were modeled using voom, and differential expression between CK2α knockout and control samples was assessed using linear modeling within the limma framework, followed by empirical Bayes moderation. *P*-values were adjusted for multiple testing using the Benjamini–Hochberg method to control the FDR, and genes with FDR < 0.05 were considered statistically significant. The resulting moderated statistics provided $\log_2$ fold changes for CK2α knockout (logFC_CK2KO).

(4) Definition of the FBL-responsive gene set and CK2α-dependent refinement

To identify genes reciprocally regulated by p53 and FBL depletion, two criteria were applied: (1) genes up-regulated in p53-KO versus WT under DOXO treatment in GSE42368 (FDR < 0.05 and Δlog_2_DOXO > 0), and (2) genes down-regulated upon *FBL* knockdown in GSE205366 (FDR < 0.05 and logFC_siFBL < 0). The intersection of these two sets defined the FBL-responsive gene set. To further assess CK2α dependency, a third criterion was applied: (3) genes down-regulated upon CK2α (CSNK2A1) knockout in U937 cells in GSE217776 (FDR < 0.05 and logFC_CK2KO < 0). The intersection of criteria (1) – (3) defined the CK2α-dependent subset of the FBL-responsive genes.

Comparison of FBL- and CK2α-associated proteomics datasets

FBL-associated proteins were retrieved from the publicly available proteomics dataset PXD034434, in which SFB-tagged FBL was affinity-purified from HCT116 cells and analyzed by nanoLC–MS/MS. CK2α-associated proteins identified in nuclear fractions were obtained from dataset PXD040882, in which FLAG-tagged CK2α complexes were immunoprecipitated from nuclear fractions of synchronized HEK293 cells during early and late G1 phases, followed by LC–MS/MS analysis. Proteins identified in either early or late G1 nuclear fractions were combined and considered CK2α-associated for subsequent analysis. Protein lists derived from the FBL and CK2α proteomics datasets were harmonized using UniProt accession identifiers, and overlaps between the two lists were determined.

The FBL–CK2α overlapping proteins were further intersected with a curated list of validated DNA G4-binding proteins compiled from previous studies ([53]; Supplementary Data 5) to identify candidate factors present in all three sets.

Analysis of endogenous G4 formation at promoter-proximal regions

Endogenous G4 formation in promoter-proximal regions was analyzed using publicly available BG4 ChIP-seq datasets. Processed BG4 peak data for HepG2 and K562 cells were obtained from the Gene Expression Omnibus (GSE145090), using the files HepG2_async_rep1-3.mult.6of9.bed and K562_async_rep1-3.mult.5of8.bed.

Transcription start site (TSS) regions were defined using the GRCh38 multi-TSS annotation, and multi-TSS coordinates corresponding to 135 genes of interest were extracted based on Ensembl gene IDs. Promoter-proximal regions were defined as ±1 kb windows centered on each TSS in a strand-independent manner.

Overlaps between BG4 peaks and the extended TSS windows were quantified using bedtools intersect in count mode. For each gene, BG4 peak counts were summed across all associated multi-TSS windows, and BG4 presence was defined as at least one overlapping peak. Genomic coordinates of BG4-positive TSS windows were deduplicated, sorted, and consolidated at the gene level using custom shell and AWK scripts.

Computational environment

All bioinformatic analyses were performed on a Linux-based system using R (version 4.3.3) with Bioconductor packages including limma (version 3.58.1) and edgeR (version 4.0.16). Genomic interval operations were performed using BEDTools, and standard Unix shell utilities were used for data handling and preprocessing.

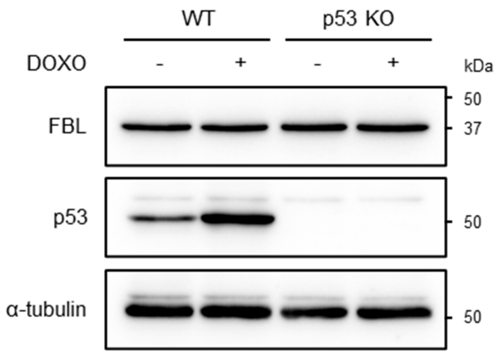

Supplementary Figure S1. FBL protein levels in WT and p53 KO HCT116 cells with or without DOXO treatment.

$p53^{+/+}$ (WT) and $p53^{-/-}$ (p53 KO) HCT116 cells were treated with or without DOXO (1 µg/mL) for 16 h. Cell lysates were subjected to immunoblot analysis with antibodies against FBL, p53, and α-tubulin as a loading control.

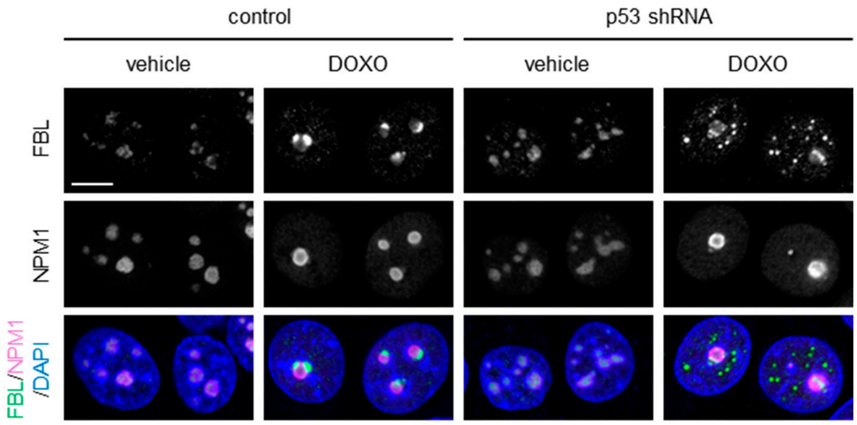

Supplementary Figure S2. The accumulation of nucleoplasmic FBL was induced by DOXO treatment in p53 knockdown MCF-7 cells.

MCF-7 cells expressing control or p53 shRNA were treated with or without DOXO (1 µg/mL) for 16 h. Confocal images of FBL (green), NPM1 (magenta), and DNA stained using DAPI (blue). The scale bar is 10 µm.

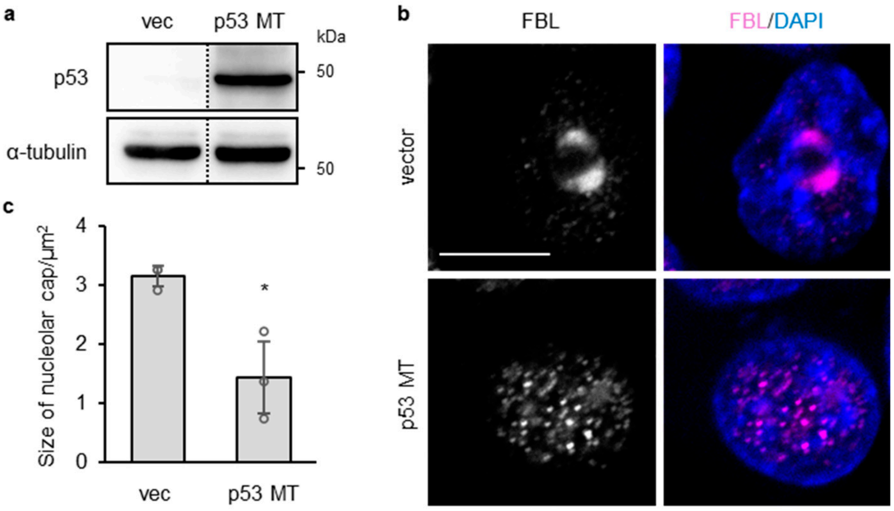

Supplementary Figure S3. Dominant-negative p53 reduces nucleolar cap formation and increases nucleoplasmic FBL localization in DOXO-treated WT HCT116 cells.

WT HCT116 cells were co-transfected with an EGFP expression vector together with either a control vector or a dominant-negative p53 mutant expression vector and then treated with DOXO (1 μg/mL) for 16 h. EGFP-positive cells were selectively analyzed to identify transfected cells. (**a**) Cell lysates were subjected to immunoblot analysis with antibodies against p53 and α-tubulin as a loading control. (**b**) Confocal immunofluorescence images of FBL (magenta) and DNA stained with DAPI (blue). The scale bar is 10 μm. (**c**) Quantification of nucleolar cap size. The area of the largest nucleolus-associated FBL-positive structure per cell was measured as the nucleolar cap, whereas FBL-positive structures with an area < 0.5 μm² were defined as nucleoplasmic FBL foci Approximately 30 EGFP-positive cells were analyzed per experiment. Data are presented as mean ± SD from three independent experiments. Statistical significance was assessed using an unpaired two-tailed t-test with Welch's correction. * indicates $p < 0.05$.

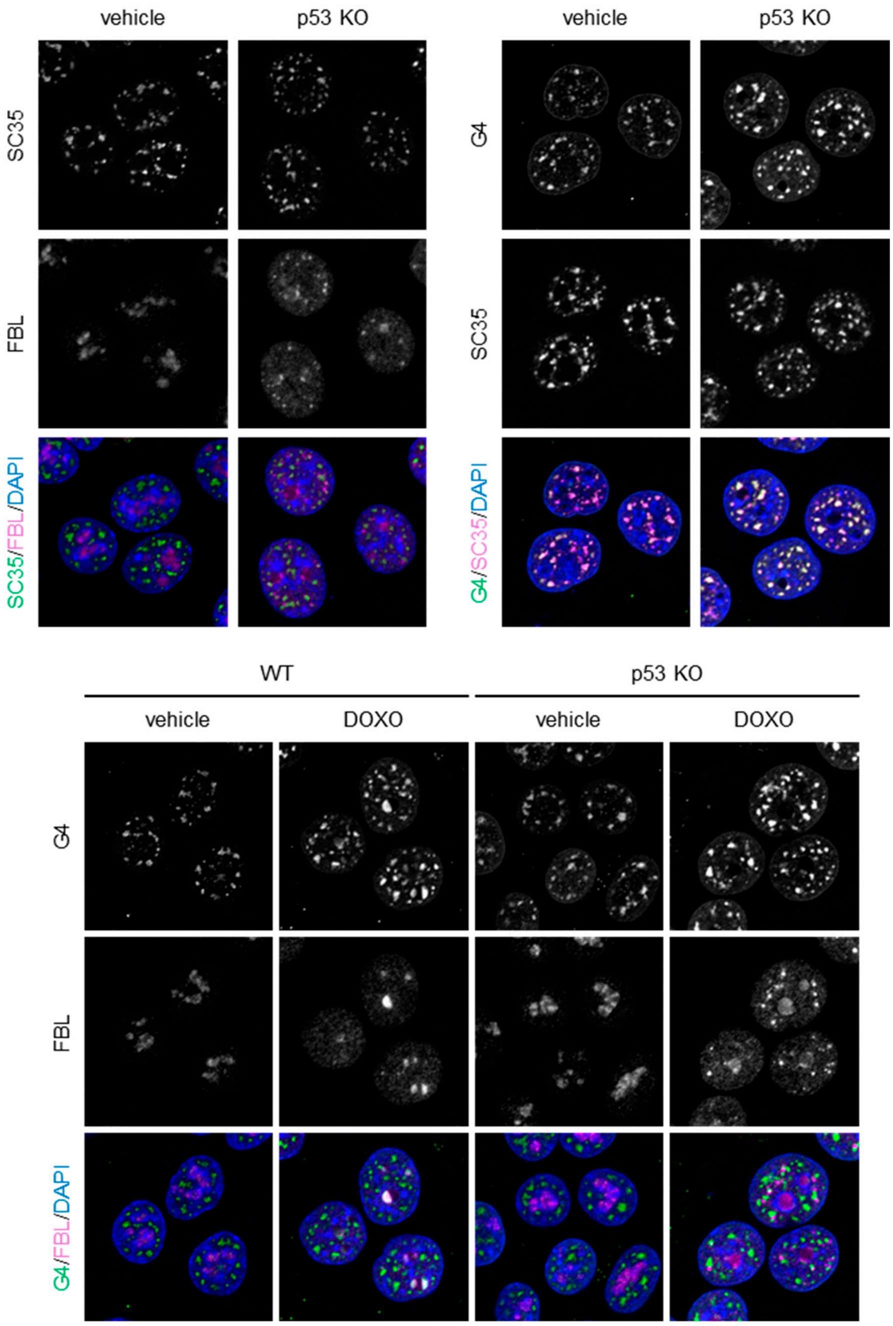

Supplementary Figure S4. Original confocal images corresponding to Figure 4.

Panels (a), (b), and (c) correspond to Figures 4a, 4c, and 4e, respectively.

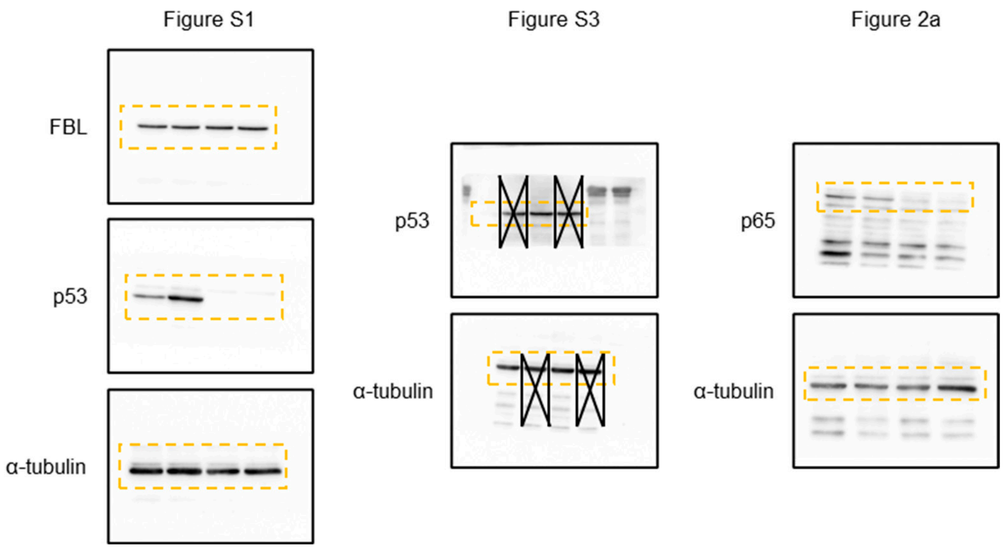

Supplementary Figure S5. Original uncropped images used for Western blot analysis.

[1] Kawauchi, K.; Araki, K.; Tobiume, K.; Tanaka, N. Activated p53 induces NF-κB DNA binding but suppresses its transcriptional activation. *Biochem Biophys Res Commun* **2008**, 372, 137-141, doi:10.1016/j.bbrc.2008.05.021.
